# The Moderating Effect of Body Appreciation on the Relationship between Self-Esteem and Life Satisfaction

**DOI:** 10.3390/ejihpe14040056

**Published:** 2024-03-28

**Authors:** René Wodarz, Aleksandra M. Rogowska

**Affiliations:** Institute of Psychology, University of Opole, 45-040 Opole, Poland

**Keywords:** adults, body image, body appreciation, life satisfaction, self-esteem, well-being

## Abstract

Background: Although positive associations between life satisfaction, self-esteem, and body image have previously been established, differences in these variables by gender and age have yielded mixed results. Moreover, little is known about the interplay between self-esteem and body appreciation on life satisfaction. This study aims to investigate the moderating effect of body appreciation on the relationship between self-esteem and life satisfaction, considering disparities between females and males and also between emerging adults (before the age of thirty) and older adults. Methods: A cross-sectional online survey was performed in Poland with a sample of 449 adults aged between 18 and 75 (*M* = 30.41, *SD* = 12.72), including 68% of women. The survey included the Satisfaction With Life Scale (SWLS), Rosenberg Self-Esteem Scale (RSES), and Body Appreciation Scale (BAS-2). Results: Men scored higher than women in terms of life satisfaction and self-esteem, while older participants (age > 30) scored higher than younger individuals (age ≤ 30) in terms of life satisfaction, self-esteem, and body appreciation. The study confirmed positive and moderate correlations between life satisfaction, self-esteem, and body appreciation. The interactive effect of self-esteem and body appreciation on life satisfaction was also found by controlling for age and gender. Conclusions: Some intervention programs focused on increasing levels of self-esteem and body appreciation should be implemented, especially among women and emerging adults, to improve their well-being.

## 1. Introduction

The assessment of one’s body image holds pivotal importance in an individual’s existence, concurrently impacting various facets related to quality of life, such as self-esteem and levels of life satisfaction [1,2]. Nonetheless, an equally significant aspect is the way individuals perceive their own appearance, considering cultural and social influences, along with the ensuing comparisons with others. The objective of this research was to investigate the associations between body appreciation, self-esteem, and life satisfaction in adults. These domains are of particular interest within the realms of positive psychology and an evolutionary psychological approach. The study aimed to pinpoint factors influencing appearance satisfaction, also examining how respondents’ gender and age might impact the overall outcomes of the analyzed variables.

Theories of adult development describe it as stages of growth in which individuals begin to see the world from a broader perspective and gain a more complex understanding of themselves and others [3,4,5]. According to Erikson’s theory of psychosocial development [3,6,7], adulthood can be divided into three periods during which it is necessary to solve the crisis, understood as a development task, in order to positively move on to the following stages of life. In early adulthood (aged 18–40), a crisis occurs as a result of the “intimacy versus isolation” conflict. The developmental task is to move from selfish thinking about oneself to caring for other people in the world and achieving the virtue of love. The next psychosocial crisis occurs in middle adulthood, between the ages of 40 and 65, and refers to the conflict between creativity and stagnation. A positive solution to this psychosocial crisis leads to the achievement of the virtue of caring. The last, eighth stage of late adulthood begins after the age of 65 and is associated with the conflict between “integrity versus despair”. The demographic changes that occurred in the second half of the 20th century resulted in an extension of the education period and an increasingly later age of marriage and parenthood. The period of young adulthood between the ages of 18 and 29 is now called emerging adulthood in developmental psychology [8]. This transition period between adolescence and adulthood includes many challenges that involve a sense of instability, exploration of one’s own identity, uncertainty about the future, and self-focus, which significantly reduce the quality of life, health, and well-being [9,10,11].

Life satisfaction, also known as “well-being” or “quality of life” [12], involves the assessment of various life domains, encompassing elements such as physical health, financial stability, job satisfaction, personal identity, and social relationships [13]. Determining life satisfaction goes beyond a simple sum or average of these factors, as some aspects may hold greater significance for individuals while others may exert only a marginal influence [14]. According to Diener [15,16], life satisfaction is the outcome of comparing one’s situation with self-established standards. Research showed a non-linear correlation between age and life satisfaction [17,18]. The study conducted by Darbonne et al. [18] illustrates that the life satisfaction trajectory with age takes on a “U” shape, with average values reaching their lowest point around the age of 47. Blanchflower and Oswald [17] also corroborate a decline in life satisfaction during midlife, with the nadir typically occurring between the ages of 40 and 49. Studies exploring the connections between life satisfaction and gender have been extensively conducted, yet the results are inconclusive and contingent on context [19,20,21].

Self-esteem is defined as the belief in one’s worth or evaluations of one’s traits and competencies and is grounded in self-awareness and the subjective opinions individuals hold about themselves [22]. According to Rosenberg [23], high self-esteem is a result of self-assessment as a valuable member of society, while low self-esteem indicates dissatisfaction with oneself. Global self-esteem represents a relatively stable attitude towards oneself, formed in early childhood and influencing partial assessments and specific areas of self-esteem [24]. Research consistently indicates that men score higher than women in self-esteem [25,26,27,28,29,30]. Research examining the impact of age on self-esteem varies depending on economic, professional, and family achievements, which is the highest in middle adulthood [31,32].

According to Cash et al. [33], body image is a complex concept that encompasses cognitive, behavioral, and emotional aspects related to overall body appearance. Satisfaction with one’s body and attention focused on it are pivotal for a sense of self-attractiveness. Shoraka et al. [34] define body image as a subjective picture of individuals of their own body, independent of the objective appearance of their physical form. Grogan [35] interprets body image as a combination of feelings, thoughts, and perceptions about one’s body, while Thomson [36] suggests that body image, or perceived attractiveness, involves a subjective perspective on one’s appearance. The author highlights the emotional component of this process and the individual’s awareness of being judged by their surroundings. The development of body image is shaped by a range of factors, including self-esteem, environmental influences, and objective attractiveness [37]. Research indicates a positive correlation between subjective self-attractiveness and the group’s objective assessment [38]. The perception of body image can be affected by the opinions of significant others, accepted social norms, prevailing beauty standards in social comparisons, and the body silhouettes commonly depicted in the media [39]. Throughout the socialization process, individuals engage in comparisons of their appearance with others [40]. Therefore, middle-aged people and men usually assess their body image as more positive than adolescents or older people and women, respectively [2,31,41,42,43].

Positive body image is understood in a multi-aspect, comprehensive, and stable way, including body appreciation, body acceptance and love, internal positivity, interpreting information in a way that protects the body, a broad conceptualization of beauty, and adaptive investment in appearance [44,45]. Positive body image is related to self-esteem and the acceptance of one’s body by others, which creates social identity [45]. Body appreciation is one of the crucial dimensions of positive body image. It is defined as accepting and holding favorable opinions about one’s body, resisting sociocultural pressures to internalize stereotypical beauty standards, and appreciating the functionality and health of the body [46,47]. Body appreciation not only acts as a safeguard against negative states, but also as a catalyst for fostering overall health and well-being [48]. Respect and a positive attitude towards one’s body are associated with various indicators of mental well-being and physical health, including optimism, self-esteem, proactive coping, positive affect, life satisfaction, subjective happiness, and emotional intelligence [44]. Research shows that individuals who cultivate a sense of appreciation for their bodies tend to be more inclined to adopt health-promoting behaviors [49,50]. Therefore, body appreciation can contribute to overall well-being [51].

Several studies within the realm of positive psychology centered on the aspects of life satisfaction, self-esteem, and the evaluation of physical attractiveness. These variables, heavily shaped by subjective appraisal, play a substantial role in shaping both self-perception and one’s outlook on the surrounding world. Two noteworthy connections deserve attention: the correlation between self-esteem and life satisfaction, and the relationship between the assessment of body image and self-esteem. In particular, the perception of one’s attractiveness plays a pivotal role in shaping overall self-esteem [52]. An individual’s appearance serves as a kind of label, subject to social judgment and comparisons with others. Numerous studies suggest a positive correlation between self-esteem and various dimensions of body image [31,52,53,54,55,56,57,58]. A considerable amount of research showed the positive associations between perceived attractiveness and life satisfaction, as well as the overall sense of life quality [54,57,59,60]. Also, previous studies indicate a positive relationship between self-esteem and life satisfaction [20,57,60,61,62,63,64]. Although positive correlations between self-esteem, body appreciation, and life satisfaction were established previously [47,51,65,66,67,68,69,70,71], the moderating effect of body appreciation was not examined in the relationship between self-esteem and life satisfaction. In particular, little is known about these associations, taking into account gender and age differences. Previous studies showed that women and emerging adults are the most vulnerable groups for decreased levels of mental health and well-being, as well as self-esteem and body image. Therefore, in this study, these populations will be under control when the potential effect of both self-esteem and body appreciation on life satisfaction will be examined for the first time. If self-esteem and body appreciation interact with life satisfaction, future prevention and intervention programs should be designed for specific targeted groups. Promoting both positive body image and self-esteem can be more effective for improving mental health and well-being than programs focused separately on body image or self-esteem, especially among women and emerging adults.

The following hypotheses will be verified based on the scientific literature presented above:

- H1: Men score higher than women in life satisfaction, self-esteem, and body appreciation.
- H2: People in middle and late adulthood present higher levels of life satisfaction, self-esteem, and body appreciation than emerging adults and those in early adulthood periods of life.
- H3: There are positive correlations between life satisfaction, self-esteem, and body appreciation.
- H4: Body appreciation plays a moderating role in the relationship between self-esteem and life satisfaction.

## 2. Materials and Methods

### 2.1. Study Design and Procedure

A cross-sectional online study was performed in Poland between 18 October and 6 December 2021. The survey was developed using Google Forms and disseminated via Facebook groups (including such groups as “Students—let’s connect”, “Surveys—I’ll be happy to help you fill in”, “We fill out surveys”, “Student surveys”, “Seniors on Facebook”, “Seniors in Wroclaw”, “Seniors in Warsaw”, and “Seniors 50+”). The study details and informed consent information were presented on the first webpage, and only those who agreed to participate completed the entire survey. The participants willingly and voluntarily participated in the research. Informed consent was obtained from all subjects involved in the study. The study was conducted in accordance with the Declaration of Helsinki and approved by the University of Opole Research Ethics Committee (Decision No. 1/2022).

An a priori power analysis was conducted using G*Power ver. 3.1.9.6 [72] to calculate the minimum number of participants needed to test the study hypotheses. The results indicated that the required sample size to achieve 80% power for detecting a medium effect (η^2^*_p_* = 0.06) for a two-way analysis of variance (ANOVA), at a significance criterion of α = 0.05, was *N* = 269. To determine the medium correlation effect (*r* = 0.30), at a significance criterion of α = 0.05 and 80% power, we needed a total sample of 67 people. To determine the medium interaction effect (η^2^*_p_* = 0.06) for two groups, at a significance criterion of α = 0.05 and 80% power, a sample of 269 people should be examined. Initially, 527 people responded to the invitation to the study, but 6 of them did not consent to participate, 17 did not meet the criteria of age (were under 18), and 5 nonbinary persons were also excluded for further statistical comparisons of women with men. The final sample included 499 participants. The post hoc analysis showed that the power exceeded 0.99% for ANOVA, Pearson’s correlation, and moderation analysis. Thus, the obtained sample size of *N* = 499 is more than adequate to test all study hypotheses.

### 2.2. Measurement

#### 2.2.1. Demographics

The demographic questions were about sex (women, men, nonbinary), age (number of years old), place of residence (village, town up to 50,000 inhabitants, a city of 50,000 up to 150 thousand inhabitants, a city from 150 thousand up to 500 thousand inhabitants, and a city of over 500,000 residents) and education (primary, vocational, secondary, Bachelor’s degree, Master’s degree, and other).

#### 2.2.2. Life Satisfaction

The Satisfaction With Life Scale (SWLS) includes five items related to the cognitive and global assessment of life satisfaction (e.g., “In most ways, my life is close to my ideal”) [15,73,74]. Participants rated on a 7-point Likert scale how much they agreed with a given sentence (from 1 = Strongly disagree to 7 = Strongly agree). Total scores range from 5 to 35, and higher scores indicate a higher level of life satisfaction. The internal consistency (Cronbach’s α) of the SWLS was 0.89 in the present study.

#### 2.2.3. Self-Esteem

The Rosenberg Self-Esteem Scale (RSES) measures a relatively stable disposition understood as a conscious attitude towards the self [23,62,75]. Participants rate their consensus on a 4-point Likert scale (1 = Strongly disagree, 4 = Strongly agree) for each of the ten items (e.g., “I take a positive attitude toward myself”). Higher scores (ranging from 10–40) indicate more positive self-esteem. The internal consistency is Cronbach’s α = 0.91.

#### 2.2.4. Body Appreciation

The second version of the Body Appreciation Scale (BAS-2) was developed to collect “favorable opinions about self-body (regardless of actual appearance), acceptance of the body despite the weight, body shape and imperfections, respect for the body by meeting its needs and engaging in healthy behaviors, and protecting the body by rejecting unrealistic body images presented in the media” [76]. We used the Polish adaptation of the BAS-2 [77]. Participants respond to each of ten items (e.g., “I feel good about my body”) on a 5-point Likert scale (1 = Never, 5 = Always). The scores range from 10 to 50, and higher scores mean a more positive attitude toward the self-body. The reliability of the BAS-2 was Cronbach’s α = 0.96 in the present study sample.

### 2.3. Participants

A sample of 499 people aged between 18 and 75 (*M* = 30.41, *SD* = 12.72), including 336 women (68%), participated in the study. Among the respondents, 7% (*n* = 37) declared receiving primary education, 3% (*n* = 17) reported vocational education, 35% (*n* = 172) reported secondary education (high school), 27% had a Bachelor’s degree (*n* = 134), and 28% (*n* = 139) had a Master’s degree. Most participants lived in the countryside (*n* = 159, 32%), 19% (*n* = 97) lived in cities with over 500,000 inhabitants, 18% (*n* = 90) lived in cities with between 50,000 and 150,000 residents, 17% (*n* = 87) lived in cities with up to 50,000 residents, and 13% (*n* = 67) of the respondents lived in cities with between 150,000 and 500,000 inhabitants. We divided the sample according to age: Younger (<30 years, *n* = 317, including 97 males and 220 females) and Older (≥30 years, *n* = 182, including 65 males and 117 females).

### 2.4. Statistical Analysis

The psychometric properties of all continuous variables (SWLS, RSES, BAS-2) were examined in regard to the range of scores, mean (*M*), standard deviation (*SD*), median (*Mdn*.), kurtosis, skewness, and Cronbach’s α reliability coefficient. Since the sample size was large (*N* = 499), and skewness and kurtosis range ± 1, violation from normal distribution should not have been a problem, and parametric statistical tests were performed to verify the hypotheses. A two-way ANOVA was performed to examine differences in life satisfaction, self-esteem, and body appreciation between the biological sexes (Female, Male) and age-related groups (Younger, Older). Pearson’s correlation analysis was conducted to test the relationships between variables. Also, multiple linear regression analysis was conducted to examine the moderating role of body appreciation in the association between self-esteem and life satisfaction. All statistical analyses were performed using IBM SPSS ver. 26. Model 1 of the PROCESS macro ver. 4.2 for SPSS was applied for moderation analysis. The bootstrap technique (with 5000 resampling and 95% confidence interval) was used to estimate the interaction effect with greater accuracy and reliability. The bootstrap method can provide bias-corrected estimates of interaction effects and accelerated confidence intervals, improving the accuracy of statistical inferences.

## 3. Results

### 3.1. Intergroup Differences in Life Satisfaction, Self-Esteem, and Body Appreciation

A two-way ANOVA was performed to examine the sex and age differences in terms of life satisfaction, self-esteem, and body appreciation (Table 1). All main effects were statistically significant for life satisfaction as a dependent variable, and factors such as sex, age, and interaction between sex and age were used in the ANOVA model. However, the effect size was small for these effects. A post hoc Bonferroni test indicated that males scored higher than females in terms of life satisfaction (*p* < 0.05), and older participants scored higher than younger ones (*p* < 0.001). In particular, older men reported higher levels of life satisfaction than younger males (*p* < 0.01) and younger females (*p* < 0.001). Among the older participants, males scored significantly higher in terms of life satisfaction than females (*p* < 0.05).

Considering self-esteem, significant main effects were found for sex and age (with a small effect size), but not for interactions between them. A post hoc Bonferroni test revealed an overall higher level of self-esteem in males than in females (*p* < 0.05) and in older than in younger participants (*p* < 0.001). In particular, younger males scored significantly lower for self-esteem than older males (*p* < 0.001) and older females (*p* < 0.05). Similarly, younger females scored lower for self-esteem than both older females and males (*p* < 0.001). In addition, a sample of older men demonstrated higher self-esteem than older women (*p* < 0.05).

There were significant main effects of sex and age on body appreciation. Generally, men scored higher for body appreciation than women (*p* < 0.05), while people over thirty years of age scored higher than their younger counterparts (*p* < 0.01). In addition, older men scored significantly higher for body appreciation than younger men (*p* < 0.05) and younger women (*p* < 0.01).

### 3.2. Associations between Life Satisfaction, Self-Esteem, and Body Appreciation

The first step in examining the associations between life satisfaction, self-esteem, and body appreciation was to perform Pearson’s correlations. Medium positive correlations were found for all variables at *p* < 0.001 (Table 2).

**Figure 1:**
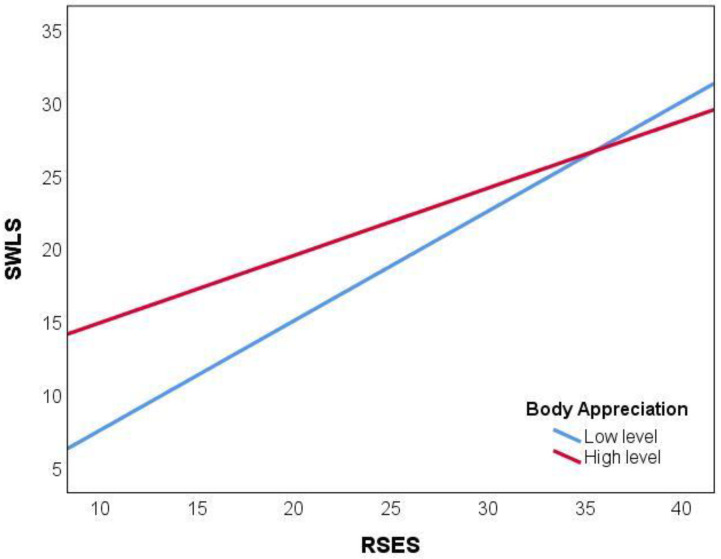
The moderating effect of body appreciation on the relationship between self-esteem (RSES) and life satisfaction (SWLS).

Multiple linear regression was performed using PROCESS ver. 4.2. software, with life satisfaction as an explained variable, self-esteem as a predictor, and body appreciation as a moderator. Sex and age were included in the regression model as confounders (Table 3). The results indicate that life satisfaction can be explained by self-esteem (*p* < 0.001) and body appreciation (*p* < 0.001). An interaction effect between self-esteem and body appreciation on life satisfaction was also found (*p* < 0.01). The effect of self-esteem on life satisfaction is stronger for people with lower levels of body appreciation compared to people who are more appreciative of their bodies (Figure 1). Age and sex were not significant predictors of life satisfaction in the regression model (Table 3). The regression model explains 53% of life satisfaction variance, with *R*^2^ = 0.53, *F* (5, 493) = 108.84, and *p* < 0.001.

## 4. Discussion

### 4.1. Sex Differences in Life Satisfaction, Self-Esteem, and Body Appreciation

The study showed that the life satisfaction, self-esteem, and body appreciation levels were higher in men than in women, but the effect size for these differences was small. As such, hypothesis H1 was fully confirmed. Previous research showed an ambiguous association between life satisfaction and gender [19,20,21]. For instance, research on Canadian and Polish adolescents did not reveal significant differences between genders [19,20]. An examination of data from the World Values Survey (WVS) highlights regional variations in the level of life satisfaction. Women report higher levels of life satisfaction in Muslim and East Asian countries, while men achieve higher scores in post-communist countries, Latin America, and European countries with a Catholic influence [21]. Meisenberg and Woodley [21] suggested that a history of communism and limited political freedom have a stronger negative impact on the subjective well-being of women than men, while long-term education appears to be more harmful to men than to women. Furthermore, these studies suggest a negative correlation between higher female gender status, greater gender equality, and subjective life satisfaction in women. Elevated social status and respect for the female gender are associated with lower subjective life satisfaction in women [21]. The authors explain that women’s higher life satisfaction in countries with traditional gender roles (e.g., Muslim and East Asian countries) may result from women’s lower expectations. Also, Western women demonstrate, on average, lower levels of ambition, competitiveness, risk-taking, and materialism than men. Wąsowicz-Kiryło and Baran [20] emphasize a strong association between life satisfaction in women, financial well-being, and job satisfaction.

Higher levels of self-esteem in men than in women were also evidenced previously [25,26,27,28,29,30]. Research conducted by Kling et al. [27] indicates that men frequently exhibit higher scores on self-esteem scales compared to women. This trend is corroborated by Kearney-Cooke [26] and other investigators, including Josephs et al. [25], Lewinsohn et al. [28], and Steinberg and Dornbusch [30]. A meta-analysis of 216 studies substantiates this connection, although the differences are relatively minor [27]. Researchers attribute these distinctions to the impact of cultural and social expectations. According to Kling et al. (1999) [27], men’s self-esteem may be a result of a self-fulfilling prophecy influenced by societal expectations, where the traditional assertive male role contributes to elevate self-esteem.

Nonetheless, alternative viewpoints suggest that it is not necessarily that men inherently possess higher self-esteem; rather, women may experience lower self-esteem, a phenomenon also influenced by cultural and social factors. McMullin and Cairney [29] highlight the correlation between self-esteem and factors such as income, social standing, and professional status. Men, who are often in managerial roles with higher earnings, tend to experience elevated self-esteem. Conversely, for women, achieving similar professional success may lead to reduced self-esteem, arising from a subjective perception of fewer opportunities for success. Research indicates that stronger emphasis is placed on the cultivation of positive self-esteem in Western cultures compared to East Asian and non-Western cultures [78,79]. Cai et al. [80] suggest that the self-enhancement motive can be determined by socialization pressures, such as cultural constraints (e.g., norms, rules, values, and inhibitions). Western culture promotes independent self-construal and individualistic attributes (e.g., original, unique) as desirable or personally important, which increases self-esteem. In contrast, Eastern culture fosters interdependent self-construal and collectivistic attributes (e.g., loyalty, respect) as desirable, which may decrease self-esteem as an important personal attribute. Eastern culture emphasizes avoidance or prevention goals and fosters concern with negativity. Therefore, lower levels of explicit self-esteem are presented in the East than in the West [80].

Consistent with previous studies [2,31,41,42,43], the current research finds a marginal effect for gender differences in body appreciation without interaction of gender with age. Men scored higher than women in terms of body appreciation, which is in line with previous studies performed among Austrian, Brazilian, German, Indonesian, Iranian, and Romanian adult populations [65,70,81,82,83,84], and university students from France [85] and Hong Kong [86]. However, no gender differences were found for body appreciation in samples of Polish [77] and U.S. adults [87], and also among older people (aged 65–91 years) from Portugal [88]. Furthermore, the BAS-2 scale was invariant across gender and country in a sample of adolescents and young adults from Denmark, Portugal, and Sweden [67]. Gender invariance was also found in the BAS-2 scale among German [84], Japanese [68], Polish [77], and Spanish adults [69], as well as among U.S. college students [46,89]. Cross-cultural differences can explain some discrepancies between studies concerning gender differences for body appreciation. Recently, Swami et al. [90] performed a multi-group confirmatory factor analysis (CFA) of the BAS-2 across 65 nations, 40 languages, and various gender identities and age groups. Higher levels of body appreciation were related to greater cultural distance from the United States and greater relative income inequality. Although significant differences across nations and languages were found in latent body appreciation, the differences across gender identities and age groups were negligible to small. In particular, body appreciation was higher in men than other gender identities (including women). Additionally, a positive correlation was confirmed between body appreciation and life satisfaction [90].

Body image may be related not only to self-esteem and gender, but also to gender roles, as suggested in previous research [40]. For example, feminine women rated their physical appearance less favorably than androgynous women, which seems to be related to the degree of cultural standards and acceptance around the importance of appearance for women. In contrast, masculinity in females correlated positively to a more favorable body image. The study among cisgender and gender minority samples also showed no significant differences in body appreciation between heterosexual and sexual minority adolescents [91]. Dignard and Jarry [92] found that body appreciation negatively correlated with body dissatisfaction and investment for aesthetic purposes among young Canadian female undergraduates. Furthermore, most items of the BAS-2 scale were likely to be interpreted in terms of appearance in young women. Indeed, exposure to body ideals in the media can play a detrimental role in developing body image disturbances [93]. Also, the solid societal idealization of the athletic body may expose people to the pressure of having a specific body appearance, which may result in unfavorable health consequences [47,94].

### 4.2. Age Differences in Life Satisfaction, Self-Esteem, and Body Appreciation

We found that older individuals (age > 30) scored higher than younger people (age ≤ 30) for life satisfaction, self-esteem, and body appreciation (but the effect size was relatively small). The findings conformed to hypothesis H2 in this study. In general, various hypotheses attempt to elucidate the relationship between life satisfaction and age. While research indicates that age plays a predominant role, other factors such as happiness, economic status, and marital status may also exert an impact, albeit to a lesser degree [95]. The reasons for this relationship remain not definitively defined, and multiple perspectives and factors appear to contribute to this intricate phenomenon. It is proposed that individuals in midlife encounter heightened stress associated with professional, social, and familial responsibilities [17]. Within the age range of 40–50, there is an elevated likelihood of interpersonal tensions linked to the formation of value systems and the endeavor to safeguard resources. Researchers suggest that this phase may mark a turning point, leading to a decrease in the perception of life quality [18]. Following the trough in life satisfaction, these individuals may become more resilient to environmental stress, adapting to circumstances and embracing their vulnerabilities. This adaptation could result in achieving financial stability and reducing both the number and significance of social roles. Older individuals might develop more effective coping strategies for managing adverse events. Additionally, there is a suggestion that society may treat older individuals more positively, contributing to the reinforcement of the life satisfaction effect [96].

In line with developmental psychology principles, older individuals may become more accepting and appreciative of themselves, contributing to an enhancement in self-esteem. Erikson [5] underscores that the later stages of life represent a period for achieving balance, affirming the meaning of life, and fostering self-acceptance. However, from the standpoint of social roles, the perspective suggests that self-esteem might decline as individuals withdraw from professional and social roles. Previous studies showed that levels of self-esteem changed across the lifespan [31,32]. An analysis of research employing the Rosenberg Self-Esteem Scale indicates variations in self-esteem based on both age and gender, with cultural influences, social roles, and professional accomplishments playing a role in shaping self-esteem [31]. Other studies propose that self-esteem may see an uptick in one’s forties, linked to economic, professional, and family achievements [32]. However, around the age of 60, a decline in self-esteem becomes apparent, possibly attributed to a shift in attitude toward a more modest and humble perspective. While middle age represents a phase of optimal life stimulation, maturity, and adaptation, self-esteem may gradually diminish in later years [32].

The present study found significant differences between younger and older adults. In particular, emerging adults scored lower in terms of body appreciation than their older counterparts. This result is consistent with previous research [84]. Tiggemann and McCourt [97] found a significant positive correlation between age and body appreciation among adult women aged between 18 and 75 years. Older women presented higher levels of body appreciation than younger participants. Similarly, a positive correlation was presented between body appreciation and age among German women aged 16–74 years [84]. In contrast, a large international study [90] indicated that adults aged 25–44 years reported slightly lower body appreciation than those aged 18–24 years and older adults (aged ≥45 years). Studies exploring the impact of age and gender on body image highlight a substantial influence of these factors on the evaluation of one’s body and the associated emotions [2,31,41,42,43]. Concerns regarding body evaluation start to surface as early as the age of two, and initial social interactions can contribute to the development of these concerns [41]. Throughout adolescence, particularly in females, shifts in perceived body image occur due to significant physical changes, potentially leading to lower attractiveness ratings [2]. In contrast, for males, the assessment of attractiveness tends to remain relatively stable, a phenomenon explained by theories of sexual selection [2]. In early adulthood, both genders may experience a decline in satisfaction with their appearance, a trend that gradually reverses in mid-adulthood. Developmental crises and the aging process can trigger renewed decreases in attractiveness ratings, often linked to signs of aging, fatigue, and social alienation [42,43].

### 4.3. Relationships between Life Satisfaction, Self-Esteem, and Body Appreciation

As assumed in H3, positive correlations were found between life satisfaction, self-esteem, and body appreciation, with medium strength (*r* ranged between 0.61 and 0.72). A positive correlation between body image and self-esteem was found previously [31,52,53,54,55,56,57,58,65]. Similar to our study, Bale and Archer [53] demonstrated moderate correlations between body attractiveness and self-esteem, reporting *r* = 0.65 for the assessment of body attractiveness. In the MSEI questionnaire [31], physical attractiveness is considered a component of self-esteem, encompassing the self-assessment of appearance and sexual attractiveness. The questionnaire includes inquiries about the overall level of attractiveness, satisfaction with one’s appearance, the frequency of receiving compliments from others, and comparisons with others. The internal correlation between overall self-esteem and physical attractiveness stands at *r* = 0.69, which is also similar to our study. Other studies [55,56] also indicate a positive relationship between the assessment of one’s own body and self-esteem. Feingold’s [55] study indicates a moderate correlation between self-rated attractiveness and self-esteem, with a more pronounced association in women (*r* = 0.32) than in men (*r* = 0.27), potentially influenced by cultural norms that emphasize the significance of women’s appearance. In a study conducted by Kochan-Wójcik and Piskorz [56] involving 476 women, a positive correlation between self-esteem and perceived body image was also confirmed. Similar findings were observed in the study conducted by Khalaf et al. [98] on a sample of 237 female and male students. They presented evidence supporting a positive correlation between higher body appreciation and elevated self-esteem, also measured by the BAS-2 and RSES scale [98]. Overall, research suggests a reciprocal relationship between self-esteem and perceived physical attractiveness, where each variable can influence the other [53,58].

The previous literature also revealed positive associations between body image and life satisfaction [54,57,59,60]. Diener et al. [59] illustrated a correlation between the subjective evaluation of attractiveness and life satisfaction (*r* = 0.29), suggesting that individuals with higher life satisfaction may also tend to have elevated self-esteem regarding their attractiveness. Cash et al. [33] validated a positive correlation between life satisfaction and body image assessment, considering variables like current body weight and preferred body weight. In a multicultural study by Lee et al. [57], a positive correlation between life satisfaction and body image assessment was affirmed among both Americans (*r* = 0.54) and individuals from Korea (*r* = 0.46). In a study by Delfabbro et al. [54] exploring the correlates of dissatisfaction with one’s attractiveness, the results indicated that lower subjective body image ratings were linked to lower life satisfaction, self-esteem, and diminished ratings of mental health. Tager et al. [99] indicated a positive correlation between perceived body attractiveness and life satisfaction, particularly in young men. However, it is crucial to acknowledge that perceived life satisfaction is influenced by various factors [99]. Studies also reveal robust correlations between body image and life satisfaction, with the association being more pronounced in the case of women [60]. Researchers underscore the moderating influence of the respondent’s body weight, which may be linked to overall health and physical fitness, subsequently impacting overall life satisfaction [60].

Consistent with previous studies [57,59,60,61,63,64], we confirmed the positive association between self-esteem and life satisfaction. The findings from Swami et al.’s [60] studies underscore the substantial role of self-esteem in the development of life satisfaction. A comprehensive review of the literature on the relationships between self-esteem and life satisfaction uncovered statistically significant correlations. However, their intensity and strength varied based on the cultural context, age and gender of the participants, and the measurement tools employed [61]. Nevertheless, certain studies underscore the moderating influence of factors like perceived quality of life or the quantity of social contacts. Campbell [61] hypothesized that self-esteem serves as a robust predictor of life satisfaction, reporting a correlation of *r* = 0.55. The research findings of Diener and Diener [62] involving a diverse group of students (*N* = 13,118) from various cultures demonstrated a moderate correlation of *r* = 0.47 between self-esteem and life satisfaction. Other investigations [20] revealed a positive correlation, with a moderate association for both men (*r* = 0.31) and women (*r* = 0.40). In addition, self-esteem carried greater significance for women in influencing life satisfaction. In research focusing on nursing home residents, the correlation between self-esteem and life satisfaction was found to be *r* = 0.25 [64]. Enhanced correlations were also noted among individuals from collectivistic cultures and in relationships characterized by support and reciprocity [63]. Lee et al.’s [57] investigations, involving a representative group of 502 Americans and 518 Koreans, also revealed a potent influence of subjective self-esteem on overall life satisfaction. The impact of self-esteem on life satisfaction was notably stronger in the American group, with cultural disparities being attributed to this observation. It was argued that positive self-esteem is deeply ingrained in North American culture, motivating citizens to possess and reinforce favorable judgments about themselves. This study also indicated the significant role of self-esteem in shaping the perception of happiness and the sense of life quality.

In the present study, the regression analysis showed that the interaction between self-esteem and body appreciation predicts life satisfaction. Consistent with hypothesis H4, we confirmed that body appreciation moderates the relationships between self-esteem and life satisfaction. The effect of self-esteem on life satisfaction is stronger for people with lower levels of body appreciation than those who better appreciate their bodies. This association is not affected by age and gender. The regression model explains 53% of life satisfaction variance. The current findings are in line with previous research [52] showing that the perception of one’s attractiveness is a pivotal factor in shaping overall self-esteem. Appearance is characterized as a kind of label subject to judgment from individuals in our environment, emphasizing that satisfaction or dissatisfaction with the assessment of one’s own body can significantly impact overall self-esteem. In the Multidimensional Self-Esteem Inventory (MSEI), subjective physical attractiveness was recognized as a contributing variable to the broader construct of self-esteem [31]. The questionnaire incorporated a subscale specifically addressing self-esteem linked to one’s appearance. The items in the questionnaire encompassed not only the direct evaluation of one’s appearance, but also factors such as comparisons with others and the frequency of receiving compliments from others [31]. This implies a notable impact of the environment on both body image and overall self-esteem. The previous findings also demonstrated that the degree of contentment with one’s appearance, as well as self-esteem, can serve as a significant predictor of life satisfaction [57]. The present study extended the previous literature by showing that there is a significant interaction between self-esteem and body appreciation, which determines life satisfaction in adults.

### 4.4. Practical Implications

The study found a moderating effect of body appreciation on the relationship between self-esteem and life satisfaction. Research suggests that people with low levels of body appreciation and low self-esteem demonstrate lower life satisfaction levels than those with higher body appreciation and low self-esteem. In contrast, people with a low level of body appreciation but high self-esteem show a significantly higher level of life satisfaction compared to those whose self-esteem and body appreciation are both high. These findings have implications for promotion and intervention programs, which should be focused simultaneously on self-esteem and body appreciation to increase well-being and mental health, particularly among women and emerging adults. Educational settings, particularly universities, can utilize the insights garnered from the study to design and implement targeted strategies aimed at cultivating positive body image among individuals [100]. By doing so, there is an opportunity to impact self-esteem levels positively. One effective approach involves integrating specialized educational courses that focus on promoting body positivity and self-acceptance. These courses can cover topics such as body diversity and mental well-being, fostering a more inclusive and supportive environment. Sundgot-Borgen et al. [47] suggested including the implementation of media literacy, body functionality, and exercise as topics within the education program that promote body appreciation and prevent body appearance pressure. The media literacy approach to body image and eating disorder risk reduction can be especially effective through improving a positive body image and embodiment lens [101,102]. In addition to educational courses, organizing inspirational campaigns within the community can further contribute to the enhancement of positive body image. These campaigns could involve awareness initiatives, workshops, and events that celebrate diversity, challenge stereotypes, and encourage a healthy and realistic perspective on body image. For example, feminist- and social-justice-informed approaches showed efficacy by increasing self-efficacy and promoting health at every size, as well as positive embodiment, especially among women [101,103]. Practices such as mindfulness or yoga can also be helpful in facilitating positive body image and embodiment [104,105,106]. Among treatment programs, compassion-based interventions [107], cognitive-dissonance-based interventions [108], emotion-focused therapy [109], and acceptance and commitment therapy [110] can be recommended to increase body appreciation, self-esteem, and well-being levels.

### 4.5. Limitation of the Study

The COVID-19 pandemic mandated remote data collection, further heightening the non-random nature of the sample. Using an online survey on Facebook with the snowball method can lead to the poor representativeness of the study group. The study employed a cross-sectional design, limiting the ability to establish causal relationships. Subsequent research endeavors could consider longitudinal designs in a representative sample of adults to explore the dynamic nature of associations between life satisfaction, self-esteem, and body appreciation over time. Another limitation pertains to the use of self-report tools to assess variables, where a reliance on subjective feelings may introduce bias contingent on respondents’ perceptions. Future considerations should encompass experimental studies to enhance measurement objectivity. Future study could also benefit from addressing various professional and social aspects such as job satisfaction, occupation, and the relationship between profession and attractiveness. Also, demographic variables such as preferred religion, the level of religiosity and spirituality, or ethnicity could be controlled in future studies. The other limitation of this study is that women prevailed over men. Previous studies have also shown that women participate in surveys more often than men [111,112,113,114]. A higher response rate in women can be related to their higher agreeableness and susceptibility to suggestion and persuasion, as well as lower assertiveness, higher social desirability bias, or a higher level of interest in the research topic compared to men. However, future research should be conducted with a gender-balanced sample. A final limitation arises from the broad age range of the study groups. Narrowing the age range would enable more diverse analyses within specific age groups. While the study’s conclusions are valuable, addressing these limitations in future research could yield more comprehensive and balanced results.

## 5. Conclusions

This study enhances our understanding of the intricate relationship between body appreciation, self-esteem, and life satisfaction. It underscores notably diminished levels of satisfaction among women and emerging adults, indicating potential areas for enhancing their quality of life. Proposed interventions, such as promoting a positive body image and mitigating societal comparisons, have the potential to influence self-esteem and body appreciation positively. Initiatives aimed at improving both body appreciation and self-esteem can contribute significantly to overall well-being. In summary, this research illuminates, for the first time, the complex interplay between self-esteem, body appreciation, and life satisfaction, providing valuable insights for the promotion of mental health and overall well-being. Additionally, these findings serve as a robust foundation for conducting further research on different age groups in diverse countries and considering additional variables such as economic status or job satisfaction.

## Figures and Tables

**Table 1:** Two-way ANOVA for gender and age differences in life satisfaction, self-esteem, and body appreciation.

Variable	Male (*n* = 162)		Female (*n* = 337)		2-Way ANOVA			
	*M*	*SD*	*M*	*SD*	Effect	*F*(1, 495)	*p*	η^2^*_p_*
**Life satisfaction**					Sex	5.88	0.016	0.012
Younger (*n* = 317)	21.40	6.28	21.13	6.19	Age	12.02	<0.001	0.024
Older (*n* = 182)	24.71	5.82	22.03	6.20	S × A	3.92	0.048	0.008
**Self-esteem**					Sex	6.21	0.013	0.012
Younger (*n* = 317)	27.30	5.92	26.94	6.12	Age	47.00	<0.001	0.087
Older (*n* = 182)	32.22	4.93	29.76	5.19	S × A	3.45	0.064	0.007
**Body appreciation**					Sex	4.55	0.033	0.009
Younger (*n* = 317)	35.35	9.98	34.95	10.97	Age	9.16	0.003	0.018
Older (*n* = 182)	40.34	9.46	36.28	10.98	S × A	3.06	0.081	0.006

Note. S × A = interaction effect between sex and age.

**Table 2:** Descriptive statistics and Pearson’s correlations for life satisfaction, self-esteem, and body appreciation (*N* = 499).

Variable	Range	*M*	*SD*	Skewness	Kurtosis	1	2
1. Life satisfaction	5–35	21.86	6.25	−0.49	−0.19		
2. Self-esteem	10–40	28.36	6.01	−0.35	0.01	0.70 ***	
3. Body appreciation	10–50	36.04	10.71	−0.38	−0.92	0.61 ***	0.72 ***

*** *p* < 0.001.

**Table 3:** Multiple linear regression analysis for life satisfaction (*N* = 499).

Variable	*b*	*SE b*	*t*	*p*	LLCI	ULCI
Constant	23.08	0.60	38.55	0.000	21.908	24.261
Self-esteem	0.54	0.05	10.50	0.000	0.440	0.642
Body appreciation	0.12	0.02	4.98	0.000	0.073	0.167
Self-esteem × Body appreciation	−0.01	0.00	−3.10	0.002	−0.012	−0.003
Age	−0.02	0.02	−1.32	0.186	−0.054	0.011
Sex	−0.25	0.42	−0.59	0.556	−1.064	0.573

Note: LLCI = lower level of confidence interval. ULCI = upper level of confidence interval.

## Data Availability

The data presented in this study are available on request from the corresponding author.
